# Intervertebral disc degenerative disease in South Africa: a case-control analysis of selected gene variants

**DOI:** 10.1007/s11033-024-09930-7

**Published:** 2024-09-17

**Authors:** Keenau Pearce, Stephanie Less, Adriaan W. Liebenberg, Mongi Benjeddou

**Affiliations:** 1 Precision Medicine Unit, Department of Biotechnology, University of the Western Cape, Cape Town, South Africa; 2 Health Collective, Panorama Healthcare Building, Cape Town, South Africa

**Keywords:** Intervertebral disc degenerative disease, Single nucleotide polymorphism, Genotype, Allele, South Africa

## Abstract

**Background:**

Intervertebral disc (IVD) degenerative disease is a multifactorial disease for which genetics plays an integral role. Several genes, and their variants, associated with the development and progression of IVD degenerative disease have been identified. While several studies have investigated these genes in Asian and European populations, no available evidence exists for the South African population. Therefore, this study aimed to investigate these parameters.

**Methods and results:**

Biological samples were collected in the form of buccal swabs from patients and DNA was extracted using a standard salt-lysis protocol. DNA purity and quantity was assessed by spectrophotometry, and subsequent genotyping was performed using the MassARRAY^®^System IPLEX extension reaction. For associations between variants and the presence of IVD degenerative disease, odds ratios (OR), confidence intervals (CI), chi-squared analysis and logistic regression was calculated. Age and sex were adjusted for, and Bonferroni’s correction was applied. This study found statistically significant associations for five of the evaluated single nucleotide polymorphisms (SNPs) with IVD degenerative disease, whereby IL-1α rs1304037 and rs1800587, ADAMTs-5 rs162509, and MMP-3 rs632478 demonstrated increased odds of a positive diagnosis for IVD degenerative disease, while decreased odds of IVD degenerative disease were seen for GDF-5 rs143383.

**Conclusion:**

To the best of our knowledge, this study represents the first of its kind to investigate the association of gene variants associated with IVD degenerative disease within the South African population. This study has shown that 5 of these gene variants were significantly associated with the presence of IVD degenerative disease, reflecting their integral roles in development and possible progression of the disease.

## Introduction

Intervertebral disc (IVD) degenerative disease and related back pain are either acute or chronic forms of disease that may be caused by various factors [1, 2]. Globally, these conditions represent a significant cause of diminished quality of life, morbidity, and mortality. Lower back pain (LBP), in particular, is a common debilitating musculoskeletal condition and affects approximately 637 million people, globally–with a lifetime prevalence of approximately 80% [2–4]. In South Africa, the prevalence of diagnosed spinal degenerative disease in the urban population is estimated to range from 48 to 78.2% [5]. Many causes for LBP exist, but IVD degeneration has been found to be a more common diagnosis among individuals who suffer from LBP than those who do not. Back pain may not always be a symptom of IVD degeneration, although it is often an early sign of degenerative spine pathologies. The IVD is among the most pivotal biological structures of the human body [6, 7].

Progressive degradation of IVD structures that leads to disruption of the homeostasis of the spine is clinically known as IVD degeneration and is often associated with severe pain [7]. IVDs are composed of fibrocartilaginous tissue that assists in maintaining stability and flexibility of the entire spine [8]. The primary role of the IVD is to connect two adjacent vertebral bodies while acting as a cushion that carries weight and pressure attributed to mechanical load. An intact and healthy IVD is comprised of three main structures: the central nucleus pulposus, the external annulus fibrosus, and endplates [9]. These structures provide the IVD with high compressive and tensile strength, support axial compression of the spine, and allow multiaxial flexibility [10].

IVD degenerative disease is a multifactorial disease often emerging due to several factors causing tissue weakening which ultimately results in pathological changes in the IVD—particularly the production of inflammatory mediators, increased apoptosis, and extracellular matrix loss [10]. These include endplate damage, nutritional deficiency, abnormal load, smoking, ageing, and genetics [10]. Genetic factors in particular have been estimated to contribute an estimated 75% to IVD degenerative disease aetiology [11, 12]. Genetic variation associated with the genes involved in processes that are related to degradation of the extracellular matrix components, apoptosis, and inflammation, have been associated with structural and functional changes within the IVD, which leads to disruption of the IVD’s metabolic activities and mechanical properties [8].

To date, several genes, and their variants, associated with the development and progression of IVD degenerative disease have been identified, including COL1A1 and COL11A1, GDF-5, CASP-3 and CASP-9, IL-1α and IL-6, ADAMTS-5, KIAA, CILP, COMT, MMP-3, and MMP-6 [7, 8, 13, 14]. Notably, associated gene variants have been extensively investigated in Asian and European populations, for which risk genotypes and alleles have been identified. It is essential to note, however, that South Africa is home to several genetically diverse population groups representing unique genetic profiles which include novel and rare variants regarding pharmaco-genetically relevant genes [15–17]. Moreover, this genomic diversity is a largely understudied domain, as compared to European populations, and within the context of IVD degenerative diseases is non-existent in the available literature. It is therefore unclear how previously identified genetic associations to IVD degenerative disease relate to the South African population and the unique genetic diversity represented therein. Therefore, this study aimed to investigate selected gene variants with the greatest level of evidence for association with IVD degenerative disease within the South African population.

## Materials and methods

### Patient data collection

All participants were briefed about the project and a consent form was completed and submitted by each participant before the experiment was conducted. All clinical data was collected by the Health Collective personnel in accordance with the Helsinki Declaration of 1975, as revised in 2016, and the Protection of Personal Information Act (POPIA) of 2013, and deidentified prior to inclusion in this study [18–20]. Ethical clearance for this study was obtained from the Senate Research Committee of the University of the Western Cape [Ethics clearance number BM 22/4/7].

### Biological Sample collection

Biological samples were collected in the form of buccal swabs from patients visiting the Health Collective, Panorama Healthcare Building, Cape Town, South Africa. All patients included in this study were confirmed to have IVD degenerative disease by magnetic resonance imaging (MRI) and/or radionuclide scans and were confirmed to not have evidence of active cancer(s), a record of spinal trauma, or trauma to the surrounding spinal structures. Convenience sampling was employed for samples collected from healthy volunteers. An ethnically mixed population was utilised for the study cohort, and ethnicities of participants were determined by self-report.

### Single nucleotide polymorphism selection

The 20 relevant genetic variants selected for this study were chosen based upon previous publications, where association was made between single nucleotide polymorphisms (SNPs) and the presence of IVD degenerative disease. The Ensembl data-base, accessed on 17 June 2023, was also used for cross-referencing the selected SNPs (http://www.ensembl.org) [21].

### DNA isolation and genotyping

Genomic DNA was isolated from buccal swabs using a standard salt-lysis protocol [22]. Samples were stored at − 20 °C. DNA was quantified using a NanoDrop™2000/ 2000c UV/VIS Spectrophotometer (Thermo Scientific, Waltham, MA, USA). The SNPs were genotyped using the MassARRAY^®^System IPLEX extension reaction (Agena Bioscience, San Diego, CA, USA). Genotypes of the selected SNP variants were determined for all the study participants (Tables 3 and 4).

### Statistical analysis

Statistical tests were selected in accordance with Clark et al. (2011) and included Hardy-Weinberg equilibrium (HWE) to assess the expected and observed genotypic distribution within the study population, odds ratio (OR) as a measure of association between variants and IVD degeneration, and logistic regression to adjust for age and sex [23]. Finally, Bonferroni’s correction was employed to control for error [23]. Statistical analysis for HWE, genotype frequencies, and allele frequencies were performed using GenALEx version 6.5 [24, 25]. For HWE, *P* < 0.05 was considered significant and thus a departure from HWE. For associations between variants and IVD degenerative disease, ORs with 95% confidence intervals, and logistic regression was calculated using IBM Statistical Package for Social Sciences (SPSS) version 26. A significance threshold of 0.05 was employed for association studies.

## Results

### Demographics and covariates

Study population demographic data are shown in Table 1, and age sex and smoking were evaluated as possible covariates. Following analysis, only age (*P* = 0.0056) and sex, i.e. female (*P* = 0.0019), were determined to be covariates within the study population (Table 1). The identified covariates were subsequently utilised in logistic regression.

### Genotype distribution and hardy-Weinberg equilibrium

Genotype and allele distribution of the 20 SNPs were determined in the study participants (Tables 3 and 4). All SNPs analysed within the study population were found to be within Hardy-Weinberg equilibrium (HWE), with p-values ranging between 0.1007 and 0.9835 (Table 2).

### Association of SNPs with IVD degenerative disease

Among the SNPs selected for this study, five displayed a significant association between IVD degenerative disease and genotype or allele prior to adjustment (Table 4). All non-significant SNPs are displayed in Table 3. The five significantly associated genes/SNPs are: IL-1α rs1304037 and rs1800587, GDF-5 rs143383, ADAMTS-5 rs162509, and MMP-3 rs632478.

Prior to adjustment, the heterozygous genotype CT, and the minor allele C of rs1304037 demonstrated significant associations with diagnosis of IVD degenerative disease [*P* = 0.0456, (OR: 2.22, 95% CI: 1.01–4.91) and *P* = 0.0476 (OR:1.77, 95% CI: 1.00-3.15) respectively] (Table 4). For rs1800587, the heterozygous genotype GA, and the minor allele A demonstrated significant associations with diagnosis of IVD degenerative disease [*P* = 0.0249, (OR: 2.50, 95% CI: 1.11–5.62) and *P* = 0.0434 (OR:1.78, 95% CI: 1.01–3.14) respectively] (Table 4). When analysed, the heterozygous genotype GA, and the minor allele A of rs143383 demonstrated significant associations with diagnosis of IVD degenerative disease [*P* = 0.0299, (OR: 0.37, 95% CI: 0.15–0.90) and *P* = 0.0474 (OR:0.58, 95% CI: 0.34–0.99) respectively] (Table 4). Next, the homozygous minor genotype CC and the minor allele C of rs162509 demonstrated significant associations with diagnosis of IVD degenerative disease [*P* = 0.0152, (OR: 3.71, 95% CI: 1.29–10.68) and *P* = 0.0112 (OR: 1.99, 95% CI: 1.16–3.40) respectively] (Table 4). Finally, the heterozygous genotype GT of rs632478 demonstrated significant associations with diagnosis of IVD degenerative disease [*P* = 0.0249, (OR: 2.70, 95% CI: 1.13–6.44)] (Table 4).

Following adjustment, only the C allele of rs1304037 remained significantly associated [*P* = 0.0459 (OR:1.99, 95% CI: 1.01–3.93) respectively] (Table 4). For rs1800587, the heterozygous genotype GA, and the minor allele A remained significantly associated following adjustment [*P* = 0.0249, (OR: 2.55, 95% CI: 1.00-6.49) and *P* = 0.0434 (OR:2.14 95% CI: 1.10–4.18) respectively] (Table 4). The homozygous minor genotype CC and the minor allele C of rs162509 remained significantly associated with IVD degenerative disease following adjustment [*P* = 0.0105, (OR: 6.47, 95% CI: 1.54–27.07) and *P* = 0.0040 (OR: 2.59, 95% CI: 1.35–4.96) respectively] (Table 4). Once adjusted, only the heterozygous genotype i.e. GT of rs632478 demonstrated a significant association with diagnosis of IVD degenerative disease [*P* = 0.0057, (OR: 5.17, 95% CI: 1.61–16.61) (Table 4).

Lastly, after Bonferroni correction, significance was maintained for the A allele of rs1800587 (*P* = 0.0498), the homozygous minor genotype CC, and the minor allele C of rs162509 (*P* = 0.0210 and *P* = 0.0080), along with the heterozygous GT genotype of rs632478 (*P* = 0.0114) (Table 4).

**Table 1:** Study population demographic data demonstrating the average age, sex and smoking status within the study population, along with significant associations for age and sex (female) with IVD degeneration

Parameter		Total patients (*n* = 117)	Diagnosed IVD degenerative disease (*n* = 67)	No IVD degenerative disease (*n* = 50)	*p*-value
Age (years; mean ± SD)	Combined	50.5 ± 18.6	58.9 ± 13.2	39.2 ± 19.0	*P* = 0.0056
Sex	Male Female	47 70	23 44	24 26	*P* = 0.1390 *P* = 0.0019
Smoking		37	15	22	*P* = 0.2498

Significance (*p* < 0.05) is shown in bold

**Table 2:** SNP information including rsID numbers, gene name, chromosomal position, location, and allelic change, all evaluated SNPs were within HWE

SNP	Gene	Chromosomal position	Location	Allele change	HWE *p*-value
rs1052576	CASP-9	1:15506048	Missense	C > T	0.9260
rs1304037	IL-1α	2:112774659	3’ UTR	T > C	0.6098
rs1420100	IL-18	2:102420542	Intron	A > C	0.2543
rs143383	GDF-5	20:35438203	5’ UTR	G > A	0.9010
rs162509	ADAMTS-5	21:26953456	Intron	G > C	0.3239
rs1676486	COL11A1	1:102888582	Missense	G > A	0.1379
rs16924573	KIAA1217	10:24315964	Intron	G > A	0.6592
rs16944	IL-1β	2:112837290	Intergenic	A > G	0.1048
rs17561	IL-1α	2:112779646	Missense	C > A	0.9750
rs17576	MMP-9	20:46011586	Missense	A > G	0.9835
rs1800012	COL1A1	17:50200388	Intron	C > A	0.3443
rs1800587	IL-1α	2:112785383	Intergenic	G > A	0.7842
rs1800795	IL-6	7:22727026	Intron	G > C	0.2240
rs20575	DR4	8:23201811	Missense	G > C	0.3416
rs2073711	CILP	15:65201874	Missense	A > G	0.2693
rs2076311	COL11A2	6:33177592	Intron	C > A	0.8490
rs2228564	COL9A2	1:40307477	Missense	C > T	0.1320
rs2856836	IL-1α	2:112774506	3’ UTR	A > G	0.7926
rs4633	COMT	22:19962712	Synonymous	C > T	0.1007
rs632478	MMP-3	11:102844950	Intergenic	G > T	0.1721

Significance (*p* < 0.05) is shown in bold

**Table 3:** Genotype and allele frequencies of 20 SNPs demonstrating no significant association to IVD degenerative disease for 15 SNPs

SNP	Genotype/ Allele	Case *n* (%)	Control *n* (%)	OR (95% CI)	*p*-value
rs1052576	CC	29 (43.9)	13 (27)	Reference	
	CT	27 (40.9)	27 (56.2)	2.23 (0.96–5.19)	*P* = 0.0625
	TT	10 (15.1)	8 (16.6)	1.78 (0.57–5.56)	*P* = 0.3180
	C	85 (64.4)	53 (55.2)	Reference	
	T	47 (35.6)	43 (44.8)	1.46 (0.86–2.51)	*P* = 0.1620
rs1420100	AA	14 (21.2)	13 (26.5)	Reference	
	AC	29 (43.9)	22 (44.8)	0.81 (0.32–2.08)	*P* = 0.6722
	CC	23 (34.8)	14 (28.5)	0.66 (0.24–1.79)	*P* = 0.4104
	A	57 (43.1)	48 (48.9)	Reference	
	C	75 (56.8)	50 (51)	0.78 (0.46–1.33)	*P* = 0.3830
rs1676486	GG	40 (61.5)	29 (61.7)	Reference	
	GA	24 (36.9)	17 (36.1)	0.98 (0.45–2.14)	*P* = 0.9536
	AA	1 (1.5)	1 (2.1)	1.38 (0.08–22.97)	*P* = 0.8227
	G	104 (80)	75 (79.7)	Reference	
	A	26 (20)	19 (20.2)	1.01 (0.52–1.96)	*P* = 0.9687
rs16924573	GG	61 (93.8)	43 (89.5)	Reference	
	AG	4 (6.1)	5 (10.4)	1.77 (0.45–6.99)	*P* = 0.4130
	G	126 (96.9)	91 (94.7)	Reference	
	A	4 (3)	5 (5.2)	1.73 (0.45–6.62)	*P* = 0.4231
rs16944	AA	13 (19.6)	11 (22.9)	Reference	
	GA	25 (37.8)	22 (45.8)	1.04 (0.39–2.79)	*P* = 0.9379
	GG	28 (42.4)	15 (31.2)	0.63 (0.23–1.75)	*P* = 0.3792
	A	51 (38.6)	44 (45.8)	Reference	
	G	81 (61.3)	52 (54.1)	0.74 (0.43–1.26)	*P* = 0.2770
rs17561	CC	40 (61.5)	24 (50)	Reference	
	CA	22 (33.8)	20 (41.6)	1.51 (0.69–3.34)	*P* = 0.3021
	AA	3 (4.6)	4 (8.3)	2.22 (0.46–10.79)	*P* = 0.3220
	C	102 (78.4)	68 (70.8)	Reference	
	A	28 (21.5)	28 (29.1)	1.5 (0.81–2.75)	*P* = 0.1905
rs17576	AA	17 (26.1)	14 (28.5)	Reference	
	AG	32 (49.2)	25 (51)	0.95 (0.39–2.29)	*P* = 0.9065
	GG	16 (24.6)	10 (20)	0.76 (0.26–2.19)	*P* = 0.6102
	A	66 (50.7)	53 (54)	Reference	
	G	64 (49.2)	45 (45.9)	0.87 (0.51–1.48)	*P* = 0.6202
rs1800012	CC	43 (70.4)	36 (75)	Reference	
	CA	17 (27.8)	12 (25)	0.84 (0.36–1.99)	*P* = 0.6979
	AA	1 (1.6)	0 (0)	0.39 (0.02–10.05)	*P* = 0.5754
	C	103 (84.4)	84 (87.5)	Reference	
	A	19 (15.5)	12 (12.5)	0.74 (0.35–1.68)	*P* = 0.5196
rs1800795	GG	32 (48.4)	31 (64.5)	Reference	
	CG	28 (42.4)	12 (25)	0.44 (0.19–1.02)	*P* = 0.0563
	CC	6 (9)	5 (10.4)	0.86 (0.24–3.11)	*P* = 0.8184
	G	92 (69.6)	74 (77)	Reference	
	C	40 (30.3)	22 (22.9)	0.68 (0.37–1.25)	*P* = 0.2171
rs20575	GG	12 (18.1)	16 (33.3)	Reference	
	GC	39 (59)	23 (47.9)	0.44 (0.18–1.09)	*P* = 0.0785
	CC	15 (22.7)	9 (18.7)	0.45 (0.15–1.37)	*P* = 0.1604
	G	63 (47.7)	55 (57.2)	Reference	
	C	69 (52.2)	41 (42.7)	0.68 (0.40–1.15)	*P* = 0.1543
rs2073711	AA	14 (22.2)	9 (18.3)	Reference	
	AG	28 (44.4)	21 (42.8)	1.17 (0.42–3.20)	*P* = 0.7650
	GG	21 (33.3)	19 (38.7)	1.40 (0.49–3.99)	*P* = 0.5205
	A	56 (44.4)	39 (39.7)	Reference	
	G	70 (55.5)	59 (60.2)	1.21 (0.70–2.06)	*P* = 0.4851
rs2076311	CC	24 (38.7)	24 (48.9)	Reference	
	CA	29 (46.7)	21 (42.8)	0.72 (0.32–1.60)	*P* = 0.4274
	AA	9 (14.5)	4 (8.1)	0.44 (0.12–1.64)	*P* = 0.2238
	C	77 (62.1)	69 (70.4)	Reference	
	A	47 (37.9)	29 (29.5)	0.68 (0.39–1.21)	*P* = 0.1959
rs2228564	CC	9 (13.8)	3 (6.2)	Reference	
	CT	20 (30.7)	19 (39.5)	2.85 (0.66–12.15)	*P* = 0.1568
	TT	36 (55.3)	26 (54.1)	2.17 (0.53–8.79)	*P* = 0.2793
	C	38 (29.2)	25 (26)	Reference	
	T	92 (70.7)	71 (73.9)	1.17 (0.64–2.12)	*P* = 0.5973
rs2856836	AA	40 (61.5)	24 (51)	Reference	
	GA	22 (33.8)	20 (42.5)	1.52 (0.69–3.34)	*P* = 0.3021
	GG	3 (4.6)	3 (6.3)	1.67 (0.31–8.93)	*P* = 0.5508
	A	102 (78.4)	68 (72.3)	Reference	
	G	28 (21.5)	26 (27.6)	1.39 (0.75–2.57)	*P* = 0.2915
rs4633	CC	21 (32.3)	16 (32.6)	Reference	
	CT	25 (38.4)	23 (46.9)	1.21 (0.51–2.86)	*P* = 0.6683
	TT	19 (29.2)	10 (20.4)	0.69 (0.25–1.89)	*P* = 0.4705
	C	67 (51.5)	55 (56.1)	Reference	
	T	63 (48.4)	43 (43.8)	0.83 (0.49–1.40)	*P* = 0.4922

OR: odds ratio; CI 95% confidence interval. Percent does not account of missing alleles at specific loci.

**Table 4:** Genotype and allele frequencies of 5 SNPs demonstrating significant association to IVD degenerative disease adjusted for age and sex

				Unadjusted		Adjusted		
SNP/ Gene	Genotype /Allele	Case *n* (%)	Control *n* (%)	OR (95% CI)	*p*-value	OR (95% CI)	*p*-value	Bonferroni corrected *p*-values
rs1304037	TT	37 (57.8)	18 (36.7)	Reference		Reference		
IL-1α	CT	24 (37.5)	26 (53)	2.22 (1.01–4.91)	*P* = 0.0456	2.29 (0.91–5.75)	*P* = 0.0773	-
	CC	4 (6.2)	5 (10.2)	2.56 (0.61–10.74)	*P* = 0.1857	3.36(0.64–17.44)	*P* = 0.1491	-
	T	98 (75.3)	62 (63.2)	Reference		Reference		
	C	32 (24.6)	36 (36.7)	1.77 (1.00-3.15)	*P* = 0.0476	1.99 (1.01–3.93)	*P* = 0.0459	*P* = 0.0918
rs1800587	GG	36 (56.9)	17 (34.6)	Reference		Reference		
IL-1α	GA	22 (33.8)	26 (53)	2.50 (1.11–5.62)	*P* = 0.0249	2.55 (1.00-6.49)	*P* = 0.0488	*P* = 0.0976
	AA	6 (9)	6 (12.2)	2.11 (0.59–7.54)	*P* = 0.2409	3.50 (0.76–16.01)	*P* = 0.1063	-
	G	96 (73.8)	60 (61.2)	Reference		Reference		
	A	34 (26.1)	38 (38.7)	1.78 (1.01–3.14)	*P* = 0.0434	2.14 (1.10–4.18)	*P* = 0.0249	*P* = 0.0498
rs143383	GG	12 (18.7)	19 (38.7)	Reference		Reference		
GDF-5	GA	36 (56.2)	21 (42.8)	0.37 (0.15–0.90)	*P* = 0.0299	0.29 (0.08–1.01)	*P* = 0.0529	-
	AA	16 (25)	9 (18.3)	0.36 (0.12–1.06)	*P* = 0.0629	0.178 (0.02–1.13)	*P* = 0.0673	-
	G	60 (46.8)	59 (60.2)	Reference		Reference		
	A	68 (53.1)	39 (39.7)	0.58 (0.34–0.99)	*P* = 0.0474	0.535 (0.28-1.00)	*P* = 0.0516	-
rs162509	GG	24 (37.5)	11 (22.4)	Reference		Reference		
ADAMTS-5	CG	30 (46.8)	21 (42.8)	1.53 (0.62–3.79)	*P* = 0.3594	2.36 (0.72–7.71)	*P* = 0.1539	-
	CC	10 (15.6)	17 (34.6)	3.71 (1.29–10.68)	*P* = 0.0152	6.47 (1.54–27.07)	*P* = 0.0105	*P* = 0.0210
	G	78 (60.9)	43 (43.8)	Reference		Reference		
	C	50 (39)	55 (56.1)	1.99 (1.16–3.40)	*P* = 0.0112	2.59 (1.35–4.96)	*P* = 0.0040	*P* = 0.0080
rs632478	GG	25 (42.6)	10 (22)	Reference		Reference		
MMP-3	GT	28 (45.9)	32 (64)	2.70 (1.13–6.44)	*P* = 0.0249	5.17 (1.61–16.61)	*P* = 0.0057	*P* = 0.0114
	TT	7 (11.4)	7 (14)	2.36 (0.67–8.36)	*P* = 0.1818	2.87 (0.66–12.52)	*P* = 0.1585	-
	G	80 (65.5)	54 (54)	Reference		Reference		
	T	42 (34.4)	46 (46)	1.62 (0.94–2.79)	*P* = 0.0803	1.81 (0.94–3.49)	*P* = 0.0740	-

OR: odds ratio; CI 95% confidence interval. percent does not account of missing alleles at specific loci. significance (*p* < 0.05) is shown in bold.

## Discussion

In this study, the genetic association of 20 possible biomarkers for the presence of IVD degenerative disease was determined. All SNPs tested were found to be within HWE and showed p-values ranging between 0.1007 and 0.9835 within the study population (Table 2). Genotype and allele distribution of the 20 SNPs were determined in study cohort (Tables 3 and 4). Among the SNPs analysed, 15 of the selected SNPs exhibited no statistically significant association with IVD degenerative disease within the study cohort (Table 3). The respective genotypes and alleles of the remaining 5 SNPs i.e. rs1304037 (CT *P* = 0.0456; C *P* = 0.0476), rs1800587 (GG *P* = 0.0249; A *P* = 0.0434), rs162509 (GG *P* = 0.0152; C *P* = 0.0112), rs632478 (GT *P* = 0.0249), and rs143383 (GA *P* = 0.0299; A *P* = 0.0474), however demonstrated significant associations between variant and IVD degenerative disease, prior to adjustment (Table 4). From our analysis, SNPs rs1304037, rs1800587, rs162509, rs632478 demonstrated increased odds of a positive diagnosis for IVD degenerative disease. Conversely, decreased odds of IVD degenerative disease were seen for rs143383.

Interleukin-1 (IL-1) is an inflammatory cytokine expressed in the IVD that is tied to the degradation of extracellular matrix components through the production of degradative enzymes, inhibition of proteoglycan resynthesis, cytokine upregulation, and through inhibition of extracellular matrix component production [7, 11, 26]. The IL-1α gene in particular is linked to an increased risk of IVD degenerative disease [7]. Hypersensitivity to IL-1α in IVD cells has been described as a significant motivator for degeneration, playing a key role in extracellular matrix metabolism and modic changes—an MRI trait associated with IVD degenerative disease [27]. To date, several IL-1α polymorphisms have been shown to be associated with IVD severity and modic changes, including the rs1800587 and rs1304037 variants [26].

The C allele of the IL-1α rs1304037 variant was reported to be associated with increased severity of IVD degenerative disease and the accompanying modic changes [26]. In this study, we have similarly shown a significant association between the rs1304037 variant and IVD degenerative disease (Table 4), particularly for the CT genotype and C allele prior to adjustment. Following adjustment, the C allele remained significantly associated with IVD degenerative disease within the study cohort. Thus, the findings presented here for rs1304037 are in accordance with those reported by Parera et al. [26]. With regard to the rs1800587 variant, the TT genotype, as compared to the CC genotype, has previously been shown to be associated with and increased risk of IVD degenerative disease in several studies [27–31]. Particularly, this association was demonstrated in a Caucasian population [27, 28], a Chinese Han population [29], and a Finnish population in studies investigating middle-aged men [30] and young girls aged 12–14 [31]. Thus, from the available literature, the C and T allele combination is the most reported, however, a G and A combination has also been described. To date, two studies have demonstrated an association with IVD degenerative disease for the G and A allelic combination for the variant in a Spanish population [32], as well as a Sri Lankan population [26]. In their studies, the A allele was shown to be associated with reduced severity of IVD degenerative disease and the accompanying modic changes [26, 32]. Presently, we similarly report the G and A allelic combination for rs1800587 within the study cohort. Unlike previous studies, however, our analysis revealed a significantly increased likelihood of IVD degenerative disease for the GA genotype and A allele (Table 4). These findings are contradictory to previous reports and thus merit further investigation within a larger study cohort. Nevertheless, the findings presented here reiterate the importance of the interleukins in IVD degenerative disease and its progression.

ADAMTS form a group of metalloproteinases possessing several important biological functions, such as extracellular matrix remodelling, procollagen processing, cell migration, and inflammatory processes [33, 34]. The binding of ADAMTS to extracellular matrix components is modulated via a thrombospondin structural domain which may lead to proteolysis [34]. ADAMTS-5 (aggrecanase-2) in particular has been identified as an important risk factor in the development of IVD degenerative disease [33]. Several studies have shown an association between the ADAMTS-5 rs162509 variant and IVD degenerative disease [35–37]. Early studies of the ADAMTS-5 rs162509 variant in the Chinese Han population reported the C and G allele combination and further showed no statistically significant association with IVD degenerative disease [38]. Interestingly, however, a later study of the same population reported a G and A allelic combination and further reported the G allele to be significantly higher in patients with IVD degenerative disease, as compared to healthy individuals [37]. Similarly, a study by Rajasekaran et al. also reported a significant association between rs165209 and the severity of IVD degenerative disease in an Indian population [36]. The present analysis demonstrated the C and G allele combination within the study cohort, and further that the CC genotype and C allele were significantly associated with the presence of IVD degenerative disease following adjustment and Bonferroni correction (Table 4). These findings are contradictory to those reported by Wu et al., however, and may indicate that the ADAMTS-5 rs165209 variant plays an integral role in the presence of IVD degenerative disease within the South African population. Based on this, further, more rigorous investigation of this variant within a larger cohort is essential.

Matrix metalloproteinases (MMPs) are the principal catabolic enzymes of the IVD and are the main mediators of extracellular matrix degradation that allow for normal remodelling and the abolishment of pathological tissues [14]. Degradation of the IVD’s extracellular matrix by MMP enzymes is important in the pathogenesis of IVD degeneration [8]. MMP-3 is reportedly one of the most significant proteoglycan-degrading enzymes [8, 14]. Specific conditions such as inflammation and mechanical loading can trigger the expression of the MMP-3 gene and the resulting IVD degeneration from this expression may, hereby, increase with time [14]. While available literature is limited, the MMP-3 rs63248 variant has been shown to play an integral role toward spinal bone mineral density and degenerative disease [39, 40]. Investigations into the role of MMP-3 rs63248 variant in IVD degenerative disease by Saberi et al. demonstrated a significant association within an Iranian population [40]. More specifically, they showed that the CC genotype was associated with a significantly increased risk of IVD degenerative disease, relative to the AA genotype, and was further suggested to be a contributing factor toward increased susceptibility within the studied population [40]. Presently, the alternate G and T allele combination was detected within the SA study cohort, for which our analysis revealed a significant association for the GT genotype (Table 4). Furthermore, the GT genotype remained significantly associated with the presence of IVD degenerative disease following adjustment and Bonferroni correction (Table 4). Accordingly, these findings add a valuable contribution to the limited body of literature associating this variant to the presence of IVD degenerative disease, and further highlight the importance of MMP-3 in the aetiology of this disease. Furthermore, these findings may be indicative of an integral role of the variant for IVD degenerative disease within the South African population. However, considering the limited size of the studied cohort, further analysis in a larger population is crucial.

Members of the growth differentiation factor (GDF) family are the most significant signalling molecules that maintain the homeostasis of the IVD, and its upregulation increases the expression of healthy cell marker genes [41]. Growth differentiation factor-5 (GDF-5), which is present in both normal and degenerated IVDs, has the capacity to regulate the composition of the extracellular matrix and plays an important role in the formation of soft tissues and the development of bones, cartilage, and ligaments [41]. The polymorphism on rs143383 is located in the 5’ non-coding region of GDF-5 gene and is thought to yield downregulation of GDF-5 gene expression, ultimately yielding an increased onset risk of IVD degenerative disease [42, 43]. The rs143883 variant has, accordingly, been shown to have a strong association with the development of hip dysplasia, osteoarthritis, and lumbar-related disease in several populations [43–47]. A study by Williams et al. linked the rs143383 variant with IVD degenerative disease, reporting a significantly increased risk of disc-space narrowing and osteophyte formation in Northern European women expressing the T allele [43]. Subsequent meta-analysis has similarly shown an association between the rs143383 variant and susceptibility to IVD degenerative disease, with the T allele conferring risk and the C allele protection [46]. A more recent meta-analysis has shown the CC genotype to confer an increased incidence of IVD degenerative disease in the Chinese Han population [47]. Present analysis of the rs143383 variant in the South African cohort reveals a G and A allelic combination for the rs143383 variant, along with a significant association with the presence of IVD degenerative disease prior to adjustment (Table 4). In particular, low ORs for the GA genotype (OR: 0.38) and A allele (OR: 0.57) were observed. These findings may be indicative of a potential protective function toward GDF-5 gene dysfunction in those expressing the G/A allelic combination, and further that GDF-5 may ultimately not be involved in the presence of IVD degenerative disease within the South African population. While interesting, adjusting for age and sex diminished the statistical significance of these observations—an occurrence likely due to the limited sample size used in this study. It is therefore imperative that the relationship of the rs143383 GDF-5 variant within the SA population be evaluated within a larger cohort. Moreover, determining if this potential protective function is unique to any specific racial group within the SA population would be beneficial.

It must also be mentioned that while several significant findings have been described in this study, the relatively small population size is a notable limitation, as this may have an impact on the statistical power. Moreover, the study utilised a mixed population and thus the influence of race toward the findings cannot be excluded. Thus, it is essential that these limitations are addressed in future studies.

## Conclusion

To the best of our knowledge, this study represents the first of its kind to investigate the association of gene variants with IVD degenerative disease within the South African population. This study has shown that 5 of these gene variants were significantly associated with the presence of IVD degenerative disease, reflecting their integral roles in the development and possible progression of the disease. For this reason, further investigation is recommended within a larger study population, taking into account the possible influence of the various racial groups within South Africa.

## Data Availability

No datasets were generated or analysed during the current study.
